# Involvement of the G-Protein-Coupled Dopamine/Ecdysteroid Receptor DopEcR in the Behavioral Response to Sex Pheromone in an Insect

**DOI:** 10.1371/journal.pone.0072785

**Published:** 2013-09-04

**Authors:** Antoine Abrieux, Stéphane Debernard, Annick Maria, Cyril Gaertner, Sylvia Anton, Christophe Gadenne, Line Duportets

**Affiliations:** 1 Université d’Angers, Laboratoire Récepteurs et Canaux Ioniques Membranaires (RCIM), UPRES-EA 2647 USC INRA 1330, SFR 4207 QUASAV, Angers, France; 2 Université Pierre et Marie Curie (UPMC)-Institut National de la Recherche Agronomique (INRA), UMR 1272 Physiologie de l’Insecte : Signalisation et Communication, Université Paris VI, Bâtiment A, Paris, France; 3 Université Pierre et Marie Curie (UPMC)-Institut National de la Recherche Agronomique (INRA), UMR 1272 Physiologie de l’Insecte : Signalisation et Communication, INRA, Versailles, France; 4 Université Paris-Sud, Service d’Enseignement de Biologie Animale, Orsay, France; University of Arizona, United States of America

## Abstract

Most animals including insects rely on olfaction to find their mating partners. In moths, males are attracted by female-produced sex pheromones inducing stereotyped sexual behavior. The behaviorally relevant olfactory information is processed in the primary olfactory centre, the antennal lobe (AL). Evidence is now accumulating that modulation of sex-linked behavioral output occurs through neuronal plasticity via the action of hormones and/or catecholamines. A G-protein-coupled receptor (GPCR) binding to 20-hydroxyecdysone, the main insect steroid hormone, and dopamine, has been identified in Drosophila (DmDopEcR), and was suggested to modulate neuronal signaling. In the male moth *Agrotis ipsilon*, the behavioral and central nervous responses to pheromone are age-dependent. To further unveil the mechanisms of this olfactory plasticity, we searched for DopEcR and tested its potential role in the behavioral response to sex pheromone in *A. ipsilon* males. Our results show that *A. ipsilon* DopEcR (named AipsDopEcR) is predominantly expressed in the nervous system. The corresponding protein was detected immunohistochemically in the ALs and higher brain centers including the mushroom bodies. Moreover, AipsDopEcR expression increased with age. Using a strategy of RNA interference, we also show that silencing of *AipsDopEcR* inhibited the behavioral response to sex pheromone in wind tunnel experiments. Altogether our results indicate that this GPCR is involved in the expression of sexual behavior in the male moth, probably by modulating the central nervous processing of sex pheromone through the action of one or both of its ligands.

## Introduction

In most animals including insects, olfaction plays an important role in vital behaviors such as the search for a sexual partner, food and shelter. In many species, the most prominent use of olfactory signals is sex pheromone communication, with males generally attracted by female-produced sex pheromones inducing stereotyped sexual behavior. Responses to such pheromones depend not only on the chemical properties of the signal but also on environmental conditions, the physiological state or previous experience of the receiver [1]–[3]. The plasticity of olfactory-guided behavior, allowing animals to adapt to their environment as a function of their physiological state, depends on functional and structural modifications of the neuronal network [4]–[6]. These neuronal changes often originate from the activational and organizational actions of hormones and neuromodulators [7]–[12].

In insects, two hormones with antagonistic roles during development, juvenile hormone (JH) and the most active form of ecdysteroids, 20-hydroxyecdysone (20 E), play an important role in regulating olfactory-guided behavior. JH influences the age-dependent behavioral and central nervous responses to sex pheromone in the moth, *Agrotis ipsilon* [13], [14]. JH also modulates behavioral and peripheral pheromone responses in the long-lived moth *Caloptilia fraxinella* [9]. The role of 20 E in the control of sex pheromone responses is less studied. 20 E is involved in adult neurogenesis in the mushroom bodies, secondary olfactory centres, and important for olfactory learning and memory in the house cricket *Acheta domesticus* [15]. Recently, 20 E was shown to modulate the behavioral response to sex pheromone in *Spodoptera littoralis* and *A. ipsilon* male moths [10], [16].

In vertebrates, the involvement of steroids in the plasticity of the olfactory system has been studied through the action of their receptors. In the mouse embryo, the steroid receptor co-activator-1 mRNA is thought to play a role in the olfactory epithelium development [17]. In addition, intracellular steroid receptors can be activated by neurotransmitters like dopamine (DA) through crosstalk and convergence of membrane-initiated signaling pathways to modulate transcriptional activity of target genes, thus bypassing the well-known ligand-dependent mechanisms of activation [18].

In insects, two types of ecdysteroid receptors have been identified, which might play a role in the modulation of nervous systems. Nuclear receptors, the ecdysone receptor (EcR) and its partner Ultraspiracle (USP), have been first described in *Drosophila melanogaster* [19]. Expression of this heterodimeric receptor complex has been recently shown in peripheral and central olfactory structures of *S. littoralis* and *A. ipsilon,* and the authors hypothesized that modulation of odor responses by 20 E is mediated by these nuclear receptors [10], [16]. In parallel, a membrane-bound G-protein-coupled receptor (GPCR), binding to both ecdysteroids and DA was identified in *D. melanogaster* (DmDopEcR), and suggested to be involved in rapid non-genomic fine-tuning of neuronal circuitry [20], [21]. This receptor shows sequence homology with vertebrate β-adrenergic receptors, and rapid effects of ligand-binding are mediated via the activation of G-protein-coupled second-messenger pathways that can modulate ion channels or protein kinase activity [22]. In particular DA binds to the receptor and activates the phosphoinositol 3 kinase pathway, whereas ecdysteroid binding activates the MAP kinase pathway. The DmDopEcR receptor has a much higher affinity for ecdysteroids compared to dopamine [20]. In the migratory locust, many downstream effectors of neurotransmitters including DopEcR were shown to be up-regulated during the development of phase traits [23].

Together with hormones, catecholamines such as DA, the second ligand of DopEcR, are known to be major actors of neuromodulation in all animals. In insects, DA plays central regulation roles, especially in neural networks controlling locomotor activity [24], [25] and many stereotyped behaviors [26], [27]. In *D. melanogaster*, dopaminergic neurons modulate pheromone responses [28] and in the honeybee, queen pheromone modifies dopamine-induced behavioral effects via direct interaction with DA receptors [29], [30].

In *A. ipsilon*, newly emerged males are sexually immature and do not respond behaviorally to the female-produced sex pheromone. Three to five days after emergence, males become sexually mature and are highly attracted by sex pheromone [13]. This increase in pheromone response with age is paralleled with an increase in the sensitivity of neurons in the primary olfactory centre, the antennal lobe (AL) [14].

Using an integrative approach, we identified here DopEcR in the brain of the noctuid moth *A. ipsilon,* and studied its potential implication in the age-dependent plasticity of the olfactory system. We first cloned *DopEcR* in *A. ipsilon* (*AipsDopEcR*), revealed its tissue-specific expression, and detected the corresponding protein within the nervous system as a function of age, in relation with pheromone responsiveness. Then, we tested the effects of *AipsDopEcR* silencing by RNA interference (RNAi) on the behavioral response of sexually mature males to sex pheromone in a wind tunnel. Finally we discuss the potential role of 20 E and DA, mediated by AipsDopEcR, in the olfactory plasticity in our moth model.

## Results

### Identification and Cloning of *AipsDopEcR* in *A. Ipsilon* Males

By taking advantage of the high degree of protein sequence conservation of DopEcR receptors, we first cloned a full-length cDNA named *AipsDopEcR* via a degenerate RT-PCR reaction from *A. ipsilon* brain mRNA combined with a strategy of 5′/3′ RACE PCR (Fig. S1). This cDNA of 2126 bp deposited in the Genbank database with accession number (KC715734) contains a putative coding region of 1037 bp, a 220 bp 5′-untranslated region (5′-UTR) and a 837 bp 3′-UTR, with a polyadenylation signal upstream of the poly(A) (Fig. S1). The open reading frame encodes 345 amino acids, predicting a 39 kDa protein as determined by using MWCALC (Infobiogen). The deduced amino acid sequence of AipsDopEcR displays the putative seven transmembrane-spanning domains that characterize the GPCR family (Fig. S1). AipsDopEcR shows 67% identity with the predicted isoform A of DmDopEcR, 68% identity with *Tribolium castaneum* DopEcR, and 68% with *Apis mellifera* isoform 1 β 2-adrenergic receptor (accession numbers in Materials and Methods).

### AipsDopEcR is Predominantly Expressed in the Central Nervous System

**Figure 1:**
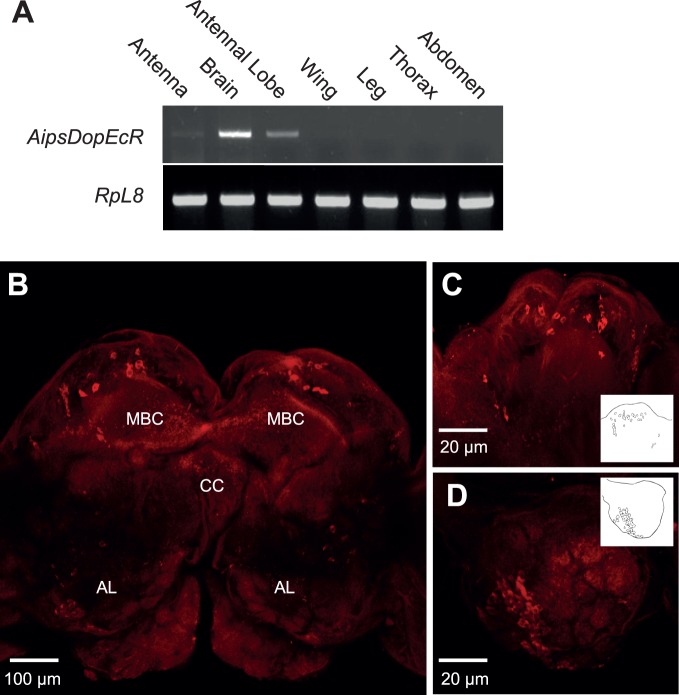
AipsDopEcR gene expression and protein detection in the brain of *A. ipsilon* males. **A)** Tissue-related *AipsDopEcR* gene expression. *AipsDopEcR* was detected mainly in the brain. **B–D)** 3D projections of confocal optical sections showing immunocytochemical AipsDopEcR protein detection in brains of 5-day-old males. **B)** Frontal view of the central brain parts. **C)** Protocerebrum with mushroom body calyx. **D)** Antennal lobe. Insets show manual reconstructions of labeled cell bodies in the respective series of sections. (MBC) mushroom body calyces; (CC) central complex; (AL) antennal lobes.

To evaluate the tissue distribution of *AipsDopEcR*, its expression was determined in the antenna, brain, AL, thorax, leg, wing and abdomen of 5-day-old males. RT-PCR analysis revealed the amplification of only one *AipsDopEcR* cDNA fragment of expected size (198 bp) whose amount was high in the brain and the AL. Only traces were detected in the antennae, and *AipsDopEcR* was not amplified in the other investigated tissues (Fig. 1A).

In whole brain immunostaining experiments with a custom-made antibody we confirmed the presence of the AipsDopEcR protein in the central nervous system in 5-day-old sexually mature males. AipsDopEcR protein expression seems to be restricted to a few areas within the *A.ipsilon* brain (Fig. 1B). We observed labeled cell bodies predominantly around the mushroom body calyces (MBC) (Fig. 1C) and in the lateral cell body cluster of the AL (Fig. 1D). We counted 30±7 labeled cells (n = 11 brains) in each MBC, and 38±8 labeled cell bodies (n = 10 brains) in each AL.

### The Expression Level of AipsDopEcR in the Brain is Age-dependent

**Figure 2:**
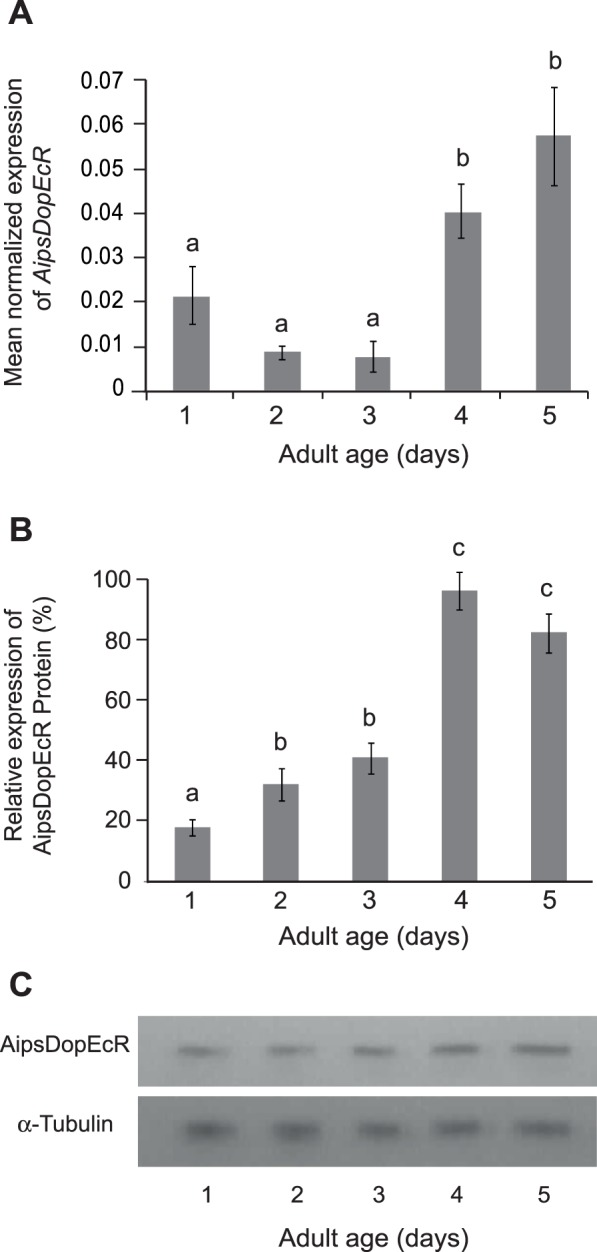
Age-related AipsDopEcR transcriptional activity and protein level from brains of 1- to 5-day-old *A. ipsilon* males. **A)** Transcriptional activity of *AipsDopEcR*. The ribosomal gene *RpL8* was used as reference. **B)** Relative protein expression of AipsDopEcR based on Western blot analysis as shown in (C). **C)** Protein expression of AipsDopEcR. The control used was the rabbit α-Tubulin protein. AipsDopEcR transcript and protein expression increase with age. Bars represent means ± s.e.m of 6 biological repetitions. Bars with same letters are not significantly different (ANOVA; Tukey test; α = 0.05).

In order to investigate if the expression of AipsDopEcR increases with age, we performed real time quantitative PCR (qPCR) and Western blot experiments on brain extracts collected from day-1 to day-5. The transcriptional level of *AipsDopEcR* was low during the first three days of adult life, and significantly higher at day-4 and day-5 (Fig. 2A).

Western blot results show that the AipsDopEcR protein is detected throughout adult life in the brain (Fig. 2B and 2C). The increased *AipsDopEcR* transcriptional activity is associated to a significant increase in brain protein synthesis at 4 and 5 days (Fig. 2B), which is correlated with the increased behavioral responses to sex pheromone [13].

### *AipsDopEcR*-dsRNA Injection Results in Reduced AipsDopEcR Expression in the Brain

To explore the role of AipsDopEcR in age-dependent pheromone-guided behavior, we used RNAi-mediated gene-silencing technology. In a first step, we evaluated the efficiency of *AipsDopEcR* gene knockdown in the brain at transcriptional and protein levels through the injection of double-stranded RNA into males (see Materials and Methods for details).

**Figure 3:**
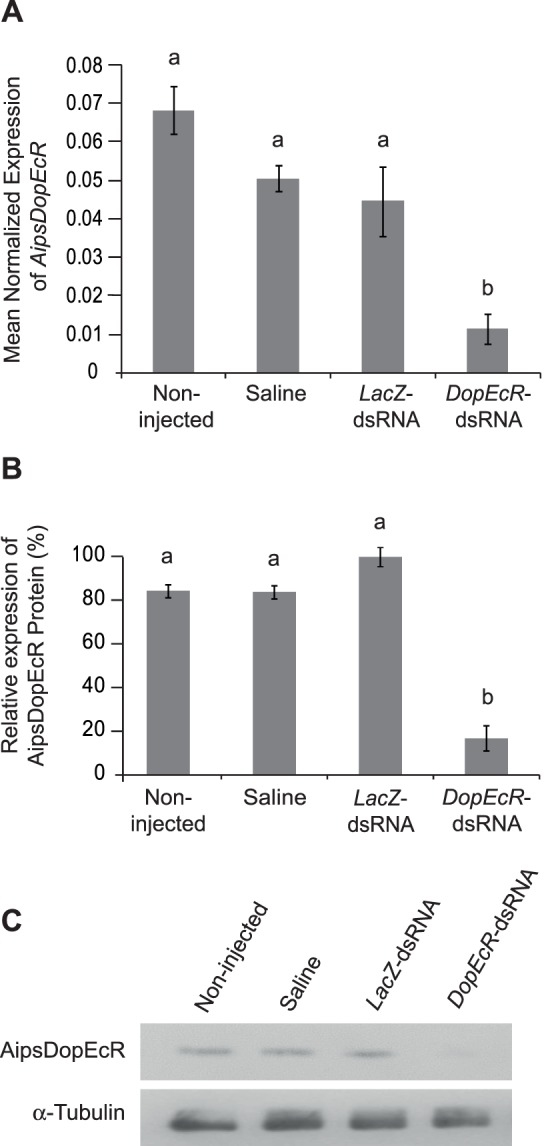
Efficiency of double-stranded RNA-mediated *AipsDopEcR* silencing in *A. ipsilon* males. 1-day-old sexually immature males received an injection of saline solution, bacterial *LacZ*-dsRNA or *AipsDopEcR*-dsRNA, or no injection (non-injected). For each treatment, the AipsDopEcR mRNA and protein amounts in the brain were evaluated 4 days after the injection by real time qPCR and Western blot respectively. **A)** Transcriptional activity of *AipsDopEcR*. The ribosomal gene *RpL8* was used as reference. **B)** Relative protein expression of AipsDopEcR based on Western blot analysis as shown in (C). **C)** Protein expression of AipsDopEcR. The control used was the rabbit α-Tubulin protein. Bars represent means ± s.e.m of 3 biological repetitions. Bars with same letters are not significantly different (ANOVA; Tukey test; α = 0.05).

*AipsDopEcR*-dsRNA administration in 1-day-old sexually immature males induced a significant decline in the amount of *AipsDopEcR* transcript 4 days after injection as compared to the three control insect groups (Saline-, Bacterial *LacZ*-dsRNA-, and non-injected males) (Fig. 3A). This transcriptional silencing was accompanied by a drastic suppression of AipsDopEcR protein synthesis in *AipsDopEcR*-dsRNA-injected males as compared with controls (Fig. 3B and 3C).

### *AipsDopEcR*-dsRNA Injection Results in Reduced Behavioral Response to Sex Pheromone

**Figure 4:**
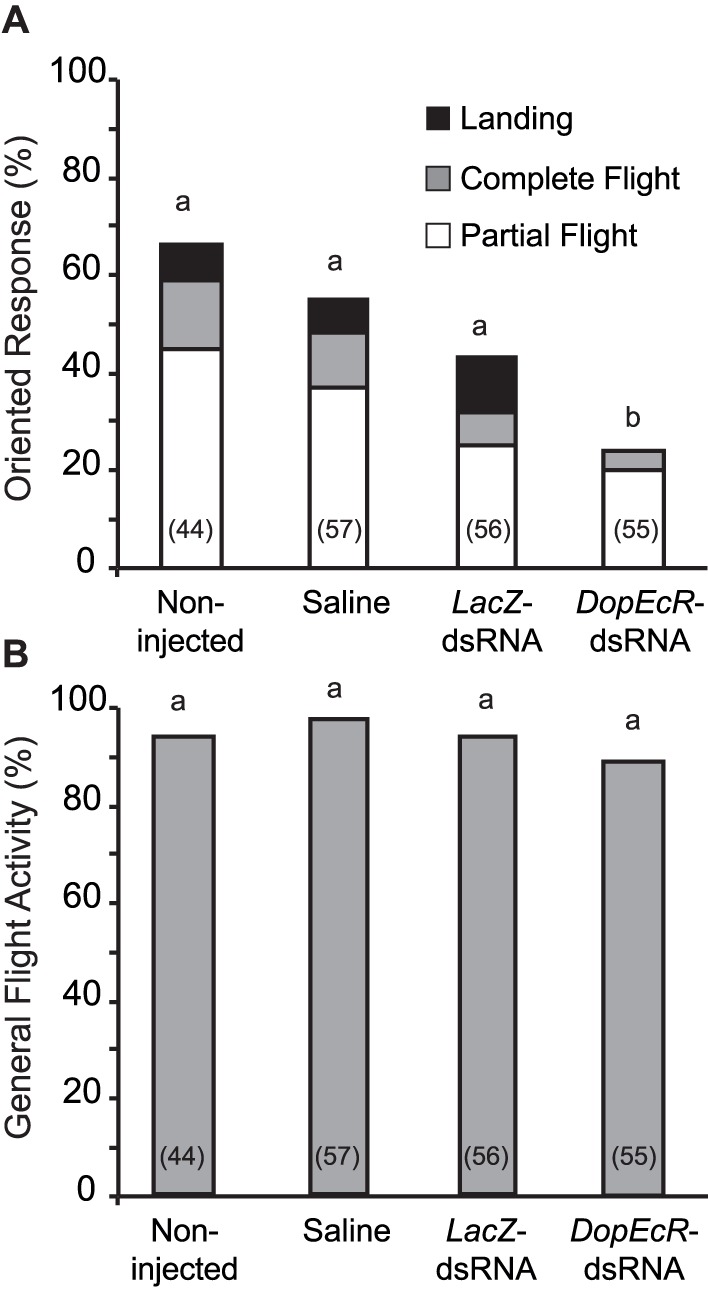
Effect of *AipsDopEcR* silencing by injection of dsRNA on the oriented responses to the sex pheromone (A) and on general flight activity (B) in *A. ipsilon* males. 1-day-old sexually immature males were injected with saline solution, bacterial *LacZ*-dsRNA or *AipsDopEcR*-dsRNA, or received no injection (non-injected). For each treatment, the percentage of oriented or random flight activity was evaluated 4 days after injection in wind tunnel experiments. **A)** Oriented responses (partial flight+complete flight+landing). *AipsDopEcR*-dsRNA injection induces a significant inhibition of oriented upwind flight towards the sex pheromone in sexually mature male moths. **B)** General flight activity (oriented responses+random flights). Locomotor behavior is not affected by *AipsDopEcR*-dsRNA injection. Numbers in brackets indicate numbers of tested males. Bars with same letters are not significantly different (G-test; P≤0.05).

In a second step, the behavioral response to sex pheromone of 5-day-old sexually mature males that were injected at day-1 with *AipsDopEcR*-dsRNA or with one of the three controls as cited above, was tested in the wind tunnel (see Materials and Methods for details). There was no significant difference in the oriented behavioral response among the three control insect groups (G-test: G = 5. 28; df = 2; p = 0.07) (Fig. 4A). However, the behavioral response of *AipsDopEcR-*dsRNA-injected males was significantly decreased as compared to that of the three control insect groups (G = 18.05; 11.18; 4.59; p<0.0001; p = 0.0008; p = 0.032; df = 1 between *AipsDopEcR*-dsRNA-injected males and non-injected, Saline-, and Bacterial *LacZ*-dsRNA-injected males respectively) (Fig. 4A). Detailed analysis shows that there is no statistical difference in the proportions of the 3 different behavioural sequences of the oriented response (partial flight, complete flight, and landing) between the four groups of insects (non-injected, saline-, lacZ-dsRNA- and DopEcR-dsRNA-injected males) (G test : G = 2.80; df = 3; p = 0.42). General flight activity was high (>90%) and statistically similar for all tested insect groups (G test: G = 2.14; df = 3; p = 0.54), thus showing that the injections did not disturb the flight performances of the moths (Fig. 4B).

## Discussion

In this study, we show the presence of AipsDopEcR, a receptor binding to 20E and DA, in the nervous system of the male moth *A. ipsilon.* Immunocytochemical detection of the corresponding protein in olfaction-related areas within the brain, changes in the levels of the receptor transcript and its protein with age, and behavioral modifications caused by silencing the receptor lead us to propose a role of AipsDopEcR in the age-dependent modulation of sex pheromone responses in this moth.

We cloned *AipsDopEcR*, the homolog of *DmDopEcR*, from nervous tissue. Its expression mainly in the central nervous system is in line with previous findings in *D. melanogaster*, in which DmDopEcR was detected essentially in heads from embryos to adults. Whereas *in situ* hybridization revealed localization of the receptor in the midgut and salivary glands in early embryonic stages of *D. melanogaster*, its late expression in development was restricted to the nervous system, suggesting a role in neuronal signaling [20]. Here we show that the AipsDopEcR protein is predominantly present in two different compartments of the brain: the MBs and the ALs, both structures involved in the processing of chemosensory cues. Ecdysteroid and DA receptors have been detected in the brain of a few insect species. In *D. melanogaster*, ecdysteroid nuclear receptors (EcR/USP) are widely expressed in the brain [31]. In the honeybee, they have been detected in the MBs and their expression changed with the foraging behavior [32]. Interestingly, EcR and USP have been detected in ALs, and their expression was recently found to be age-dependent in *A. ipsilon* [10]. DA receptors have also been detected in MBs of the honeybee [33], and in MBs, central complex and cell bodies of putative neurosecretory cells of *D. melanogaster* [34]. Also in vertebrates, such as rodents, steroid receptors have been shown to be present in both the main and accessory olfactory systems [35], and DA receptors are present in glomeruli of the olfactory bulb [36].

Our results show that levels of AipsDopEcR transcript and protein are high in the brain of sexually mature males as compared with that of young males, in correlation with a change in sex pheromone responsiveness. In certain cell types (large cell bodies) of the MBs of honeybee workers, the D-2 DA receptor expression also increased with age, thus suggesting a role in the behavioral maturation from in-hive activity to foraging [33]. Recently, DA receptors were found to be age- and behavior-regulated in the antennae of the honeybee [37]. In the migratory locust, *DopEcR* was detected in the transcriptome, and was found to be up-regulated together with other neurotransmitter receptors in gregarious as compared to solitary locusts, thus suggesting an implication in phase-related plasticity [23].

The anatomical distribution of AipsDopEcR and age-dependent changes in its expression lead us to the hypothesis that this receptor could be involved in age-dependent changes in sex-pheromone guided behavior in male *A. ipsilon.* Indeed, silencing of *AipsDopEcR* with an RNAi approach inhibited upwind flight of moths towards the sex pheromone in sexually mature males. Similarly, DopEcR, expressed in sugar-sensing gustatory receptor neurons in *D. melanogaster*, is required for the DA-mediated effect of starvation to enhance proboscis extension behavior [38]. Also in the fruitfly, the DopR1 dopamine receptor is required in the MBs for courtship learning, which reflects an enhanced response to the male pheromone *cis*-vaccenyl acetate [28], and male-male courtship [39]. In *Caenorhabditis elegans*, presence of a D2-like dopamine receptor DOP-3 is necessary for the octanol-avoidance behavior [40].

In vertebrates, various steroid- and dopamine receptors have been shown to play a role in modulation of odor-mediated behavior. In the ferret, treatment with an androgen receptor agonist increased attraction for volatile anal scent odors [41]. In mice, oestrogen receptor alpha is essential for female-directed odor-linked behavior [42], and for sexual differentiation of responses to odors [43]. The DA receptor D-2 modulates perceived odor intensity in rats [44]. In rats and mice, the D-2 receptor mediates presynaptic inhibition of olfactory neurons and its absence causes deficits in odor discrimination [45], [46].

In mammals, steroid receptors such as membrane-associated estrogen receptors are coupled with intracellular signaling pathways, which in turn allow rapid modulation of synaptic function and are therefore thought to play an important role in the fine-tuning of neuronal circuitry [21]. Ligand binding to the fruitfly DmDopEcR has been shown to elicit intracellular cAMP responses in a heterologous expression system [20].

In the noctuid moth, *A. ipsilon*, evidence is accumulating that the modulation of pheromone responses occurs through neuronal plasticity [6]. We previously showed that the male is able to gradually « switch on » its olfactory system: the behavioral and central nervous responses to female sex pheromone are age- and hormone-dependent [13], [14]. Moreover, we showed that the male can also rapidly « switch off » its olfactory system: the behavioral and central nervous responses to sex pheromone are mating-dependent and JH-independent [47]–[49]. These two forms of olfactory plasticity originate in the AL: the sensitivity of projection neurons to pheromone changes with age and mating status [14], [48]. Here we show that AipsDopEcR can modulate the behavioral response to pheromone. This class of membrane-bound receptors is known for its rapid non-genomic action, where second messenger levels can be modulated without any changes in gene expression or protein synthesis [22], [50]. It is therefore tempting to hypothesize that AipsDopEcR could act, via one or both of its ligands, on the sensitivity of AL neurons to pheromone through modulation of synaptic function.

## Materials and Methods

### Insects and Tissue Collection

Adults of *A. ipsilon* originated from a laboratory colony established in Bordeaux and transferred to Versailles. The colony is based on field catches in southern France and wild insects are introduced each spring. Insects were reared on an artificial diet [51] in individual cups until pupation. Pupae were sexed, and males and females were kept separately in an inversed light/dark cycle (16 h light: 8 h dark photoperiod) at 22°C. Newly emerged adults were removed from the hatching containers every day and were given access to a 20% sucrose solution *ad libitum*. The day of emergence was considered as day-0.

All tissue dissections were performed at mid-scotophase when males respond maximally to the sex pheromone [49]. For *AipsDopEcR* cloning and tissue/age-related expression, legs, wings, brains, ALs, antennae, thorax and abdomen of males were dissected under Ringer’s solution, then immediately deep frozen in liquid nitrogen and then stored at −80°C until treatment. For the collection of ALs, brains were first dissected, then ALs were cut from the protocerebrum with a pair of fine scissors under saline solution, and immediately dipped in Eppendorf vials kept in liquid nitrogen, then stored at −80°C.

### RNA Isolation and cDNA Synthesis

Total RNAs were extracted with TRIzol reagent (TRI Reagent®, Euromedex) according to the manufacturer’s instructions, and were quantified by spectrophotometry at 260 nm. DNase treatment was performed (2 Units TURBO™ DNase, Ambion) during 30 min at 37°C followed by an inactivation of 10 min at 75°C. After DNase treatment, single stranded cDNAs were synthesized from total RNAs (1 µg) with SuperScript II Reverse Transcriptase (Invitrogen) according to the manufacturer’s instructions.

### Cloning of *A. ipsilon DopEcR*

Degenerate DNA primers were designed on the basis of conserved amino acid sequences of *Apis mellifera* (accession number XP_396491.1), *Drosophila melanogaster* isoform A (accession number NP_647897.2) and *Tribolium castaneum* (accession number XP_968380.1) DopEcRs.

PCRs were carried out with 200 ng of brain cDNA with 2.5 units of High Expand Fidelity DNA polymerase (Roche). The degenerate primers DAEcd dir (5′- TGGAGGCBCTCAYSMAGGC-3′) and DAEcd rev (5′- GCCATRTTSCCCCASTCSAGCAT-3′) were added thereafter at 0.4 µM and each dNTP at 0.25 mM. Following an initial 5 min denaturation at 94°C, the thermal amplification procedure included 35 cycles of denaturation at 94°C for 30 s, annealing at 65°C for 30 s, elongation at 72°C for 30 s and then final elongation at 72°C for 10 min.

The 5′ and 3′ regions of the corresponding cDNA were obtained by 5′- and 3′-RACE (SMART RACE cDNA Amplification Kit, Clontech) following the manufacturer’s instructions. For 5′-RACE, we used a specific reverse primer DopEcR5′-RACE (5′- CCCCAGTCCAGCATGCATATAAAC-3′) and Universal Primer Mix (UPM, Clontech) as the forward anchor primer. The 3′-RACE amplification was carried out with UPM as the reverse primer and a specific forward primer DopEcR 3′-RACE (5′- GGAAAATGTCTTTCGACGGAAGTC-3′). Touchdown PCR was performed using hot start as follows: after 1 min at 94°C, five cycles of 30 s at 94°C and 3 min at 68°C, then five cycles of 30 s at 94°C, 30 s at 66°C and 3 min at 72°C, then 25 cycles of 30 s at 94°C, 30 s at 64°C and 3 min at 72°C, then 10 min at 72°C.

PCR products were purified by agarose gel electrophoresis (NucleoSpin® Extract II, Macherey-Nagel GmbH & Co. KG, Düren, Germany) and cloned into PCRII -Topo plasmid (Invitrogen, Carlsbad, CA, USA). After colony isolation, DNA minipreps were prepared (NucleoSpin® Plasmid DNA Purification, Macherey-Nagel GmbH & Co. KG, Düren, Germany) and the DNA clone containing the proper insert was then sequenced (GATC Biotech SARL, Marseille, France). By merging the overlapping sequences obtained from the 5′- and 3′-RACE, a putative full-length cDNA of 2126 bp was generated and named *AipsDopEcR*.

### PCR and qPCR

PCR was performed on 75 ng of cDNA preparations from various tissues with High Expand Fidelity DNA polymerase (Roche). Specific primers DopEcR dir (5′-CGTTGACCGTTATCTAGCGTTCG-3′) and DopEcR rev (5′-TTATATGCATGCTGGACTGGGG -3′) were added at 0.2 µM, and each dNTP at 0.2 mM to perform PCR reactions. Following an initial 3 min denaturation at 94°C, the thermal amplification procedure included 35 cycles of denaturation at 94°C for 30 s, annealing at 65°C for 30 s, elongation at 72°C for 30 s, and then final elongation at 72°C for 10 min. Amplification products (198 pb) were loaded on 1.5% agarose gels and visualized with Gel Red.

Real-time qPCR was done on cDNA from brains using the SYBR green detection system and LightCycler 480 Real-Time PCR System (Roche) in the technical platform of The Integrative Biology Institute (University Pierre et Marie Curie, France). Tissues were isolated from 1-day- to 5-day-old males, and RNA was extracted as described previously. Data were analyzed with Light Cycler iQ software and geNORM Visual Basic application for Microsoft Excel [52]. The standard curve was analyzed for all primers and gave amplification efficiencies of 90–100%. The control used was the *A. ipsilon* ribosomal protein RpL8 (accession number JX975720.1) whose expression was previously analyzed to be invariant considering the age of the moth. The expression of *AipsDopEcR* was normalized to geometric means of this reference and the normalized gene expression was then calculated with Q-Gene software [53].

The sequences for specific primers of *Aips-RpL8* are: Aips-RpL8dir: 5′-CCAGTTTGTCTACTGCGGCAA-3′, and Aips-RpL8rev: 5′-GCTTAACCCTAGTACGCTTGGCA-3′.

### Protein Extraction and Western Analysis

Tissues were ground with a Polytron in 100 µl of lysis buffer: 50 mM Tris–HCl (pH 7.4), 150 mM NaCl, 1% Triton X-100, 1 mM 4-(2-Aminoethyl) benzenesulfonyl fluoride hydrochloride (AEBSF) supplemented with Complete Mini Protease Inhibitors (1 tablet per 10 ml of lysis buffer, Roche). The homogenate was centrifuged during 10 min, 10,000 rpm at 4°C, and the supernatant, which contained the protein extract was quantified using the BCA assay method [54]. Samples with equal protein contents (10 µg) were adjusted to an equal volume with 50 mM Tris (pH 6.8), containing 2% SDS, 8% glycerol and 2% 2-mercaptoethanol with Bromophenol Blue as a marker, and then boiled for 3 min. Protein extracts were separated by 10% SDS-PAGE and were transferred to nitrocellulose membranes (Whatman® Protran® BA85, pore size 0.45 µm), using an electrophoretic transfer system (BioRad). Membranes were saturated 1 h at room temperature with TBS-T (Tris 1 M pH 7.4, NaCl 5 M, Tween 20) 5% Milk powder (Régilait) then incubated overnight at 4°C and 1 h at room temperature with the following primary antibodies: polyclonal anti-DopEcR raised in rabbit against the C-terminal peptide motif CD18: CRSKGRLAAELLGLDNDD of *A. ipsilon* (1∶10,000, Proteogenix) or monoclonal mouse anti-α-Tubulin (1∶640,000, Sigma T5168 isotype IgG-1). Two washes of 20 min were performed with TBS-T and the respective secondary antibody diluted in TBS-T containing 2.5% Milk powder 1 h at room temperature followed by two washes. Immune complexes were detected using a horseradish peroxidase-conjugated secondary antibody (1∶10,000 anti-rabbit IgG for DopEcR (Sigma A0545) and 1∶10,000 anti-mouse IgG for anti-α-Tubulin (Sigma A2304)). The reaction was visualized, and revealed in a dark room by 6 min exposure on photosensitive film (GE Healthcare ECL Amersham Hyperfilm™). For each Western blot α -Tubulin was used as control.

### Immunostaining

Paraformaldehyde-fixed (4%) whole brains were incubated 5 days in primary AipsDopEcR antibody diluted 1∶500 in phosphate buffered saline containing Triton X (PBST) at room temperature. After washing in PBST, the secondary antibody (Alexa fluor 555 goat anti-rabbit IgG) diluted 1∶150 in PBST was added for 4 days at 4°C. Finally samples were washed in PBST and cleared and mounted in Vectashield mounting medium (Vector Laboratories, ABCYS, France). Whole-mount fluorescent preparations were scanned with a confocal laser scanning microscope (Leica TCS SPE, Leica Microsystems, Heidelberg, Germany) using a HC PL APO CS 10.0×objective under an excitation wavelength of 555 nm to generate an emission peak at 565 nm. Partial 3D projections of optical sections were prepared using ImageJ (National Institute of Health, Bethesda, MD, USA) software. Numbers of immuno-stained cell bodies were determined manually by scrolling through stacks of optical sections.

### dsRNA Synthesis, Injection and Validation

For preparation and injections of dsRNA, and for the choice of the time delay between injections and dissections/tests, we followed the protocol used to prepare a dsRNA targeting a neuropeptide present in another moth species, *Spodoptera frugiperda* [55]. The double stranded RNA designed against *DopEcR* (586 bp) or *LacZ* (372 bp) were produced with MEGAscript® T7 High Yield Transcription Kit (Ambion) according to manufacturer’s instructions. A PCR was performed on 1 µL of plasmid (50 ng/ µL) with specific primers of each target gene DopEcR T7 dir/DopEcR T7 rev and LacZ T7 dir/LacZ T7 rev under the following program: 35 cycles of 95°C 30 s, 60°C 30 s, 72°C 1 min. The sequences of the four specific primers are:

DopEcR T7 dir: 5′-taatacgactcactatagggTAGCAGTTCGGAAGCCTCTC-3′;

DopEcR T7 rev: 5′-taatacgactcactatagggTATGTCTGACGGCGTGTTGT-3′;

LacZ T7 dir: 5′-taatacgactcactatagggATGACCATGATTACGCCAAGC-3′, and.

LacZ T7 rev: 5′-taatacgactcactatagggCCATTCGCCATTCAGGCTGCG-3′.

PCR products were purified with Nucleospin® extract II kit (Macherey Nagel) and quantified by spectrometry. Then the transcription reaction was realized at 37°C overnight on 1 µg of PCR product in a reaction mix containing 2 µL of each nucleotide, 2 µL of T7 RNA polymerase enzyme and 2 µL T7 10X Buffer for a final volume of 20 µL. After spectrometry quantification and gel dock analysis, the reaction mix was incubated during 15 min at 37°C with 2 units of Turbo DNase (Ambion). dsRNA precipitation was performed by addition of 30 µL DEPC water and 20 µL LiCl, stored at−20°C during 2 h, and centrifuged 30 min at 16,000 g. The pellet was washed with 1 mL ethanol 75%, centrifuged and dried before elution in 11 µL DEPC water. Samples were denaturated at 95°C during 5 min followed by a rehybridization step of 1 h 30 min at room temperature. Then, dsRNA integrity was checked by loading on agarose gel. Before injection, dsRNA was diluted at 0.5 µg/ µL in saline solution (CaCl2 7.1 mM, Na2 β-glycerophosphate 22 mM, MgSO4 13.5 mM, MgCl2 26.9 mM, KCl 29.5 mM, Glucose 23.9 mM, pH 6.8). qPCR and Western blot analysis were performed on treated samples in order to validate silencing efficiency.

A first series of 1-day-old adult males were injected with 1 µg dsRNA into the abdomen, and brains were dissected at day-5 for cDNA synthesis and protein extraction to validate treatment efficiency by qPCR and Western blot analysis. A second series of 1-day-old males were injected as described above, and their behavioral response to sex pheromone was tested at day-5 in a wind tunnel. For both series of experiments, control groups consisted of bacterial *LacZ*-dsRNA-, Saline-, and non-injected males.

### Wind Tunnel Experiments

Behavioral tests were performed using a 2 m-long flight tunnel during the middle of the scotophase (4–7 h after lights off) under red light illumination as previously described [49]. Environmental conditions during the bioassay were held constant: 22°C, 50±10% relative humidity, wind speed of 0.3 ms^–1^. A cage containing a single experimental male was introduced in the wind tunnel. After 30 s, during which the male adjusted to the airflow, a filter paper containing the stimulus was placed 160 cm upwind from the cage. Pheromone stimulation was performed with an artificial pheromone blend containing (Z)-7-dodecen-1-yl acetate (Z7–12:OAc), (Z)-9-tetradecen-1-yl acetate (Z9–14:OAc), and (Z)-11-hexadecen-1-yl acetate (Z11–16:OAc) (Sigma Aldrich, Saint-Quentin Fallavier, France) at a ratio of 4∶1∶4 [56], [57]. Ten ng of pheromone blend were used for all behavioral tests as this dose was shown to give the best behavioral results with sexually mature virgin males [49]. The behavior of the moths (5-day-old *AipsDopEcR*-dsRNA-injected and control males) was observed for 3 min, and partial flight (half of the distance between the source and the cage), complete flight (within 2 cm of the source) and landing on the pheromone source were considered as an oriented response towards the pheromone. Oriented as well as random flights were counted altogether in order to quantify the general flight activity of insects.

### Statistical Analysis

AipsDopEcR expression level means were compared using one-way ANOVA followed by the post hoc Tukey test if results were significant with the significance level α = 0.05. For behavioural experiments, statistical differences between groups of injected experimental males were evaluated using a *R X C* test of independence by means of a G-test and applying the Williams’s correction [58]. Regarding the statistical comparison of behavioral sequences of the 4 groups of insects, we grouped two behavioural sequences (complete flight+landing) of the AipsDopEcR-dsRNA-treated group because there was a 0 value for “landing” for this group.

## Supporting Information

Figure S1 **Nucleotide and deduced amino acid sequences of** ***A. ipsilon*** **DopEcR (AipsDopEcR).** Nucleotide (upper line) and amino acid (lower line) numbers are given on the left and on the right. The putative polyadenylation signal (AATAAA) in the 3′-UTR is indicated in italics. TM 1–7 indicate positions of transmembrane-spanning domains. The peptide sequence indicated in red corresponds to the peptide motif CD18 used for the production of the antibody.
(EPS)

## References

[1] Kolb B, Whishaw IQ (1998) Brain plasticity and behavior. Annu Rev Psychol 49: 43–64. 9496621 10.1146/annurev.psych.49.1.43

[2] Meinertzhagen IA (2001) Plasticity in the insect nervous system. Adv Insect Physiol 28: 84–167.

[3] Anton S, Evengaard K, Barrozo RB, Anderson P, Skals N (2011) Brief predator sound exposure elicits behavioral and neuronal long-term sensitization in the olfactory system of an insect. Proc Natl Acad Sci USA 108: 3401–3405. 21300865 10.1073/pnas.1008840108 PMC3044404

[4] Lledo P-M, Alonso M, Grubb MS (2006) Adult neurogenesis and functional plasticity in neuronal circuits. Nat Rev Neurosci 7: 179–193. 16495940 10.1038/nrn1867

[5] Groh C, Lu Z, Meinertzhagen IA, Rössler W (2012) Age-related plasticity in the synaptic ultrastructure of neurons in the mushroom body calyx of the adult honeybee *Apis mellifera* . J Comp Neurol 520: 3509–3527. 22430260 10.1002/cne.23102

[6] Anton S, Dufour MC, Gadenne C (2007) Plasticity of olfactory-guided behaviour and its neurobiological basis: lessons from moths and locusts. Ent Exp Appl 123: 1–11.

[7] Elekonich MM, Robinson GE (2000) Organizational and activational effects of hormones on insect behavior. J Insect Physiol 46: 1509–1515. 10980296 10.1016/s0022-1910(00)00101-3

[8] Jarriault D, Barrozo RB, de Carvalho Pinto CJ, Greiner B, Dufour MC, et al (2009) Age-dependent plasticity of sex pheromone response in the moth, *Agrotis ipsilon*: Combined effects of octopamine and juvenile hormone. Horm Behav 56: 185–191. 19409391 10.1016/j.yhbeh.2009.04.005

[9] Lemmen J, Evenden M (2009) Peripheral and behavioral plasticity of pheromone response and its hormonal control in a long-lived moth. J Exp Biol 212: 2000–2006. 19525425 10.1242/jeb.030858

[10] Duportets L, Maria A, Vitecek S, Gadenne C, Debernard S (2013) Steroid hormone signaling is involved in the age-dependent behavioral response to sex pheromone in the adult male moth *Agrotis ipsilon* . Gen Comp Endocrinol 186: 58–66. 23474331 10.1016/j.ygcen.2013.02.024

[11] Hull EM, Muschamp JW, Sato S (2004) Dopamine and serotonin: influences on male sexual behavior. Physiol Behav 83: 291–307. 15488546 10.1016/j.physbeh.2004.08.018

[12] Yoshikage M, Toshiaki I, Seiichi K, Nobuo K, Nishimura M (2007) Sex steroids modulate the signals from volatile female odors in the accessory olfactory bulb of male mice. Neurosci Lett 413: 11–15. 17125926 10.1016/j.neulet.2006.11.025

[13] Gadenne C, Renou M, Sreng L (1993) Hormonal control of sex pheromone responsiveness in the male black cutworm, *Agrotis ipsilon* . Experientia 49: 721–724.

[14] Anton S, Gadenne C (1999) Effect of juvenile hormone on the central nervous processing of sex pheromone in an insect. Proc Natl Acad Sci USA 96: 5764–5767. 10318958 10.1073/pnas.96.10.5764 PMC21934

[15] Cayre M, Strambi C, Strambi A, Charpin P, Ternaux JP (2000) Dual effect of ecdysone on adult cricket mushroom bodies. Eur J Neurosci 12: 633–642. 10712643 10.1046/j.1460-9568.2000.00947.x

[16] Bigot L, Shaik HA, Bozzolan F, Party V, Lucas P, et al (2012) Peripheral regulation by ecdysteroids of olfactory responsiveness in male Egyptian cotton leaf worms, *Spodoptera littoralis* . Insect Biochem Mol Biol 42: 22–31. 22044719 10.1016/j.ibmb.2011.10.003

[17] Misiti S, Koibuchi N, Bei M, Farsetti A, Chin WW (1999) Expression of steroid receptor coactivator-1 mRNA in the developing mouse embryo: a possible role in olfactory epithelium development. Endocrinology 140: 1957–1960. 10098538 10.1210/endo.140.4.6782

[18] Mani SK, Portillo W, Reyna A (2009) Steroid hormone action in the brain: cross-talk between signalling pathways. J Neuroendocrinol 21: 243–247. 19187467 10.1111/j.1365-2826.2009.01844.x

[19] Yao TP, Forman BM, Jiang Z, Cherbas L, Chen JD, et al (1993) Functional ecdysone receptor is the product of EcR and Ultraspiracle genes. Nature 366: 476–479. 8247157 10.1038/366476a0

[20] Srivastava DP, Yu EJ, Kennedy K, Chatwin H, Reale V, et al (2005) Rapid, nongenomic responses to ecdysteroids and catecholamines mediated by a novel Drosophila G-protein-coupled receptor. J Neurosci 25: 6145–6155. 15987944 10.1523/JNEUROSCI.1005-05.2005 PMC6725065

[21] Srivastava DP, Waters EM, Mermelstein PG, Kramár EA, Shors TJ, et al (2011) Rapid Estrogen Signaling in the Brain: Implications for the Fine-Tuning of Neuronal Circuitry. J Neurosci 31: 16056–16063. 22072656 10.1523/JNEUROSCI.4097-11.2011 PMC3245715

[22] Losel RM, Falkenstein E, Feuring M, Schultz A, Tillmann H-C, et al (2003) Nongenomic steroid action: controversies, questions, and answers. Physiol Rev 83: 965–1016. 12843413 10.1152/physrev.00003.2003

[23] Chen S, Yang P, Jiang F, Wei Y, Ma Z (2010) De novo analysis of transcriptome dynamics in the migratory locust during the development of phase traits. PLoS ONE 5: e15633. 21209894 10.1371/journal.pone.0015633 PMC3012706

[24] Akasaka S, Sasaki K, Harano K-i, Nagao T (2010) Dopamine enhances locomotor activity for mating in male honeybees (*Apis mellifera* L.). J Insect Physiol 56: 1160–1166. 20303974 10.1016/j.jinsphys.2010.03.013

[25] Mustard JA, Pham PM, Smith BH (2010) Modulation of motor behavior by dopamine and the D1-like dopamine receptor AmDOP2 in the honey bee. J Insect Physiol 56: 422–430. 19945462 10.1016/j.jinsphys.2009.11.018 PMC2834802

[26] Riemensperger T, Isabel G, Coulom H, Neuser K, Seugnet L, et al (2011) Behavioral consequences of dopamine deficiency in the Drosophila central nervous system. P Natl Acad Sci Usa 108: 834–839. 10.1073/pnas.1010930108 PMC3021077 21187381

[27] Fukumitsu Y, Irie K, Satho T, Aonuma H, Dieng H, et al (2012) Elevation of dopamine level reduces host-seeking activity in the adult female mosquito *Aedes albopictus* . Parasites Vectors 5: 92. 22574823 10.1186/1756-3305-5-92 PMC3433307

[28] Keleman K, Vrontou E, Krüttner S, Yu JY, Kurtovic-Kozaric A, et al (2012) Dopamine neurons modulate pheromone responses in Drosophila courtship learning. Nature 489: 145–149. 22902500 10.1038/nature11345

[29] Beggs K, Glendining KA, Marechal NM, Vergoz V, Nakamura I, et al (2007) Queen pheromone modulates brain dopamine function in worker honey bees. P Natl Acad Sci USA 104: 2460–2464. 10.1073/pnas.0608224104 PMC1892986 17287354

[30] Beggs KT, Mercer AR (2009) Dopamine receptor activation by honey bee queen pheromone. Curr Biol 19: 1206–1209. 19523830 10.1016/j.cub.2009.05.051

[31] Schwedes C, Tulsiani S, Carney GE (2011) Ecdysone receptor expression and activity in adult Drosophila melanogaster. J Insect Physiol 57: 899–907. 21507325 10.1016/j.jinsphys.2011.03.027

[32] Velarde RA, Robinson GE, Fahrbach SE (2006) Nuclear receptors of the honey bee: annotation and expression in the adult brain. Insect Mol Biol 15: 583–595. 17069634 10.1111/j.1365-2583.2006.00679.x PMC1847479

[33] Humphries MA, Mustard JA, Hunter SJ, Mercer AR, Ward V, et al (2003) Invertebrate D-2 type dopamine receptor exhibits age-based plasticity of expression in the mushroom bodies of the honeybee brain. J Neurobiol 55: 315–330. 12717701 10.1002/neu.10209

[34] Kim Y-C, Lee H-G, Seong C-S, Han K-A (2003) Expression of a D1 dopamine receptor dDA1/DmDOP1 in the central nervous system of Drosophila melanogaster. Gene Expr Patterns 3: 237–245. 12711555 10.1016/s1567-133x(02)00098-4

[35] Moffatt CA (2003) Steroid hormone modulation of olfactory processing in the context of socio-sexual behaviors in rodents and humans. Brain Res Rev 43: 192–206. 14572914 10.1016/s0165-0173(03)00208-x

[36] Gutièrrez-Mecinas M, Crespo C, Blasco-Ibáñez JM, Gracia-Llanes FJ, Marqués-Marí AI, et al (2005) Distribution of D2 dopamine receptor in the olfactory glomeruli of the rat olfactory bulb. Eur J Neurosci 22: 1357–1367. 16190891 10.1111/j.1460-9568.2005.04328.x

[37] McQuillan HJ, Barron AB, Mercer AR (2012) Age- and behaviour-related changes in the expression of biogenic amine receptor genes in the antennae of honey bees (*Apis mellifera*). J Comp Physiol 198: 753–761. 22930400 10.1007/s00359-012-0745-y

[38] Inagaki HK, Ben-Tabou de-Leon S, Wong AM, Jagadish S, Ishimoto H, et al (2012) Visualizing neuromodulation in vivo: TANGO-mapping of dopamine signaling reveals appetite control of sugar sensing. Cell 148: 583–595. 22304923 10.1016/j.cell.2011.12.022 PMC3295637

[39] Liu T, Dartevelle L, Yuan C, Wei H, Wang Y, et al (2008) Increased dopamine level enhances male-male courtship in Drosophila. J Neurosci 28: 5539–5546. 18495888 10.1523/JNEUROSCI.5290-07.2008 PMC6670629

[40] Ezak MJ, Ferkey DM (2010) The C. elegans D2-like dopamine receptor DOP-3 decreases behavioral sensitivity to the olfactory stimulus 1-octanol. PLoS ONE 5: e9487. 20209143 10.1371/journal.pone.0009487 PMC2830454

[41] Woodley SK, Baum MJ (2003) Effects of sex hormones and gender on attraction thresholds for volatile anal scent gland odors in ferrets. Horm Behav 44: 110–118. 13129482 10.1016/s0018-506x(03)00126-0

[42] Wersinger SR, Rissman EF (2000) Oestrogen receptor alpha is essential for female-directed chemo-investigatory behaviour but is not required for the pheromone-induced luteinizing hormone surge in male mice. J Neuroendocrinol 12: 103–110. 10718905 10.1046/j.1365-2826.2000.00418.x

[43] Bodo C, Rissman EF (2007) Androgen receptor is essential for sexual differentiation of responses to olfactory cues in mice. Eur J Neurosci 25: 2182–2190. 17419752 10.1111/j.1460-9568.2007.05484.x

[44] Wei CJ, Linster C, Cleland TA (2006) Dopamine D(2) receptor activation modulates perceived odor intensity. Behav Neurosci 120: 393–400. 16719703 10.1037/0735-7044.120.2.393

[45] Ennis M, Zhou FM, Ciombor KJ, Aroniadou-Anderjaska V, Hayar A, et al (2001) Dopamine D2 receptor-mediated presynaptic inhibition of olfactory nerve terminals. J Neurophysiol 86: 2986–2997. 11731555 10.1152/jn.2001.86.6.2986

[46] Tillerson JL, Caudle WM, Parent JM, Gong C, Schallert T, et al (2006) Olfactory discrimination deficits in mice lacking the dopamine transporter or the D2 dopamine receptor. Behav Brain Res 172: 97–105. 16765459 10.1016/j.bbr.2006.04.025

[47] Duportets L, Dufour MC, Couillaud F, Gadenne C (1998) Biosynthetic activity of corpora allata, growth of sex accessory glands and mating in the male moth *Agrotis ipsilon* (Hufnagel). J Exp Biol 201: 2425–2432. 9679104 10.1242/jeb.201.16.2425

[48] Gadenne C, Dufour MC, Anton S (2001) Transient post-mating inhibition of behavioural and central nervous responses to sex pheromone in an insect. Proc R Soc London B 268: 1631–1635. 10.1098/rspb.2001.1710 PMC1088787 11487411

[49] Barrozo RB, Gadenne C, Anton S (2010) Switching attraction to inhibition: mating-induced reversed role of sex pheromone in an insect. J Exp Biol 213: 2933–2939. 20709921 10.1242/jeb.043430

[50] Evans P, Srivastava D, Reale V (2009) Rapid, non-genomic responses to ecdysteroids and catecholamines mediated by a novel Drosophila G-Protein-Coupled Receptor. In: Smagghe G, editor. Ecdysone: Structures and Functions. Heidelberg: Springer. 425–443.

[51] Poitout S, Buès R (1974) Elevage de plusieurs espèces de lépidoptères sur milieu artificiel simplifié. Ann Zool Ecol anim 2: 79–91.

[52] Vandesompele J, De Preter K, Pattyn F, Poppe B, Van Roy N, et al (2002) Accurate normalization of real-time quantitative RT-PCR data by geometric averaging of multiple internal control genes. Genome Biol 3: research0034.0031. 12184808 10.1186/gb-2002-3-7-research0034 PMC126239

[53] Simon P (2003) Q-Gene: processing quantitative real-time RT–PCR data. Bioinformatics 19: 1439–1440. 12874059 10.1093/bioinformatics/btg157

[54] Smith PK, Krohn RI, Hermanson GT, Mallia AK, Gartner FH, et al (1985) Measurement of protein using bicinchoninic acid. Anal Biochem 150: 76–85. 3843705 10.1016/0003-2697(85)90442-7

[55] Griebler M, Westerlund SA, Hoffmann KH, Meyering-Vos M (2008) RNA interference with the allatoregulating neuropeptide genes from the fall armyworm *Spodoptera frugiperda* and its effects in the JH titer in the hemolymph. J Insect Physiol 54: 997–1007. 18541256 10.1016/j.jinsphys.2008.04.019

[56] Gemeno C, Haynes KF (1998) Chemical and behavioral evidence for a third pheromone component in a north american population of the black cutworm moth, *Agrotis ipsilon* . J Chem Ecol 24: 999–1011.

[57] Picimbon JF, Gadenne C, Bécard JM, Clément JL, Sreng L (1997) Sex pheromone of the french black cutworm moth, *Agrotis ipsilon* (Lepidoptera:Noctuidae): identification and regulation of a multicomponent blend. J Chem Ecol 23: 211–230.

[58] Sokal RR, Rohlf FJ (1995) Biometry: The principles and practice of statistics in biological research; Freeman WH, editor. New York. 315 p.

